# Randomized controlled trial comparing the effectiveness of mass and spaced learning in microsurgical procedures using computer aided assessment

**DOI:** 10.1038/s41598-021-82419-6

**Published:** 2021-02-02

**Authors:** Wendy Z. W. Teo, Xiaoke Dong, Siti Khadijah Bte Mohd Yusoff, Soumen Das De, Alphonsus K. S. Chong

**Affiliations:** 1 Department of Hand and Reconstructive Microsurgery, National University Health System, 1E Kent Ridge Rd, Singapore, 119228 Singapore; 2 Department of Orthopaedic Surgery, Yong Loo Lin School of Medicine, National University of Singapore, Singapore, Singapore

**Keywords:** Health occupations, Medical research

## Abstract

Spaced-learning refers to teaching spread over time, compared to mass-learning where the same duration of teaching is completed in one session. Our hypothesis is that spaced-learning is better than mass-learning in retaining microsurgical suturing skills. Medical students were randomized into mass-learning (single 8-h session) and spaced-learning (2-h weekly sessions over 4 weeks) groups. They were taught to place 9 sutures in a 4 mm-wide elastic strip. The primary outcome was precision of suture placement during a test conducted 1 month after completion of sessions. Secondary outcomes were time taken, cumulative performance, and participant satisfaction. 42 students (24 in the mass-learning group; 18 in spaced-learning group) participated. 3 students in the spaced-learning group were later excluded as they did not complete all sessions. Both groups had comparable baseline suturing skills but at 1 month after completion of teaching, the total score for suture placement were higher in spaced-learning group (27.63 vs 31.60,p = 0.04). There was no statistical difference for duration and satisfaction in either group. Both groups showed an improvement in technical performance over the sessions, but this did not differ between both groups. Microsurgical courses are often conducted in mass-learning format so spaced learning offers an alternative that enhances retention of complex surgical skills.

## Introduction

Microvascular surgery involves manipulation and suturing of blood vessels that range from 0.5 to 1 mm. A high degree of dexterous skill needs to be acquired prior to attempting clinical microsurgery. Currently, most surgical training courses are done over a few consecutive days, with participants practicing the skill repeatedly and continuously during this short period of time, which is a form of mass learning. However, following the course, there is decay in the acquired skills if they do not have consistent exposure to microsurgery^1^. This provides an impetus to search for alternative ways of training that are as effective and efficient in improving skill retention and clinical translation.

Spaced learning offers a viable alternative, and spacing effect refers to the phenomenon where information distributed over intervals of time (instead of being given in a bolus in a single session) enhances learning efficiency and retention^2,3^. Spaced learning has been shown to facilitate skill acquisition; short term and long term retention in motor skill training^4,5^; and may achieve better retention of skills in laparoscopy, bag-valve mask ventilation, interosseous insertions and chest compressions^6^^,^^7^. Microsurgical suturing with the use of a microscope is a far more complex and intricate task compared to suturing without visual aids, requiring fine motor coordination and delicate movements. It is also a form of a sequence task, unlike the motor adaptation tasks being examined in the aforementioned studies. If comparable or better learning outcomes and retention of information for learning of complex skills such as microsurgical suturing can be achieved with spaced learning, this may change the way training programs and course are planned. The aim of this study was to compare spaced and mass learning in microsurgery training. The null hypothesis was that there would be no difference between the two groups with respect to retention of microsurgical suturing skills.

## Materials and methods

42 medical students with no prior exposure to microsurgical training were recruited for the study in a tertiary hospital in Singapore. Students who previously underwent any microsurgical courses of any duration were excluded. Upon enrolment, participants filled in a brief survey that included baseline demographic information, hand dominance, preferences for surgical specialties and previous suturing exposure. Upon recruitment, the participants were randomized into two parallel groups in a 1:1 ratio using a computer-generated randomized number list with no restrictions. The list was only made known to the co-investigators of the study, who were responsible for generating the random allocation sequence, enrollment, and assignment of interventions. In view of the nature of how the teaching was carried out in different arms, it was not possible for the participants or instructor to be blinded. There were no changes to proposed methodology after trial commencement. The study was carried out from January–March 2017 with recruitment taking place from September–December 2016. The study was carried out in accordance with relevant guidelines and regulations and all experimental protocols were approved by the National Healthcare Group Domain Specific Review Board. Informed consent was obtained from all participants. There was no funding received throughout the course of the study. The trial has also been registered at clinicaltrials.gov (NCT03626025).

### Mass learning group

An eight-hour microsurgical suturing course was developed for the study, with a single instructor teaching participants how to handle microsurgery instruments and suture a prefabricated a standardized wide elastic strip under the microscope using MicroTrainer Platform (Digital Surgical Pte Ltd). The first two hours were spent watching an introductory video to microsurgery and familiarizing the participants with microsurgical instruments and suturing techniques. Over the next 6 h, the participants had hands-on practice and were instructed to place uniformly spaced sutures on latex strips of standard size using the microsurgical instruments given. All participants completed a single practice strip before proceeding to complete 3 strips during the single session. We placed greater emphasis on accuracy and precision of suture placement, rather than speed. The mass learning group learnt continuously over eight hours in a single session.

### Spaced learning group

Instead of undergoing a single 8-h session, the spaced learning group underwent 2-h weekly sessions for 4 weeks. The content covered was identical in both groups and was delivered by the same instructor. The participants completed a single practice strip before proceeding to complete 3 strips over the subsequent weekly sessions. Once again, we placed greater emphasis on accuracy and precision.

Additional assessments were then carried out at 1 week and 1 month after completion of the sessions in both groups to compare extent of retention of skills. Therefore, each participant performed a total of five elastic strips during the entire exercise. Strips 1, 2 and 3 were done during the teaching sessions, while Strips 4 and 5 were done at 1 week and 1-month after completion of the sessions, respectively. Strip 1 was designated as the baseline attempt, and the primary outcome of interest was the total score achieved on the final test strip (strip 5) that was performed 1 month after completion of all the sessions.

### Outcomes assessment

The primary outcome was the comparative performance (total score) of both groups at 1 month after the sessions were completed (strip 5). A computer program from Digital Surgicals was used to assess the strips—the algorithm is based on uniform and optimal suture density as well as alignment of the sutures with a maximum total score of 35 points. The method of assessment has previously been validated and eliminates the possibility of bias arising from a non-blinded assessor^8^. The secondary outcomes were time taken to complete the test strip, cumulative skills accruement, and satisfaction scores. The participants in both groups were also asked to rank their satisfaction with the structure of the course on a scale of 1–10, with 10 being highly satisfied and 1 being completely unsatisfied. The trial was deemed complete after the tests at 1 month following the end of teaching sessions. We used independent t tests to compare the total scores and duration for strip 5. Baseline characteristics like age, gender, and baseline microsurgical performance (scores from strip 1) were compared using chi-square (categorical variables) or t tests (continuous variables). Prior to statistical analysis, we examined the histograms of continuous variables to determine normality of the variables, before using the parametric tests. Finally, we performed a 2 (strips) × 2 (assigned group) mixed-model ANOVA to examine the global performance of the cohort during the actual training sessions. The within-subjects variable was strips performed over the sessions (strips 2, 3 and 4) and the between-subjects (independent) variable was the assigned group (mass versus space learning). A Bonferroni correction was applied for multiple comparisons. We used a significance level of 0.05 for the statistical comparisons. Statistical analysis was performed using SPSS (Version 21.0. Armonk, NY: IBM Corp).

## Results

### Participant flow

**Figure 1:**
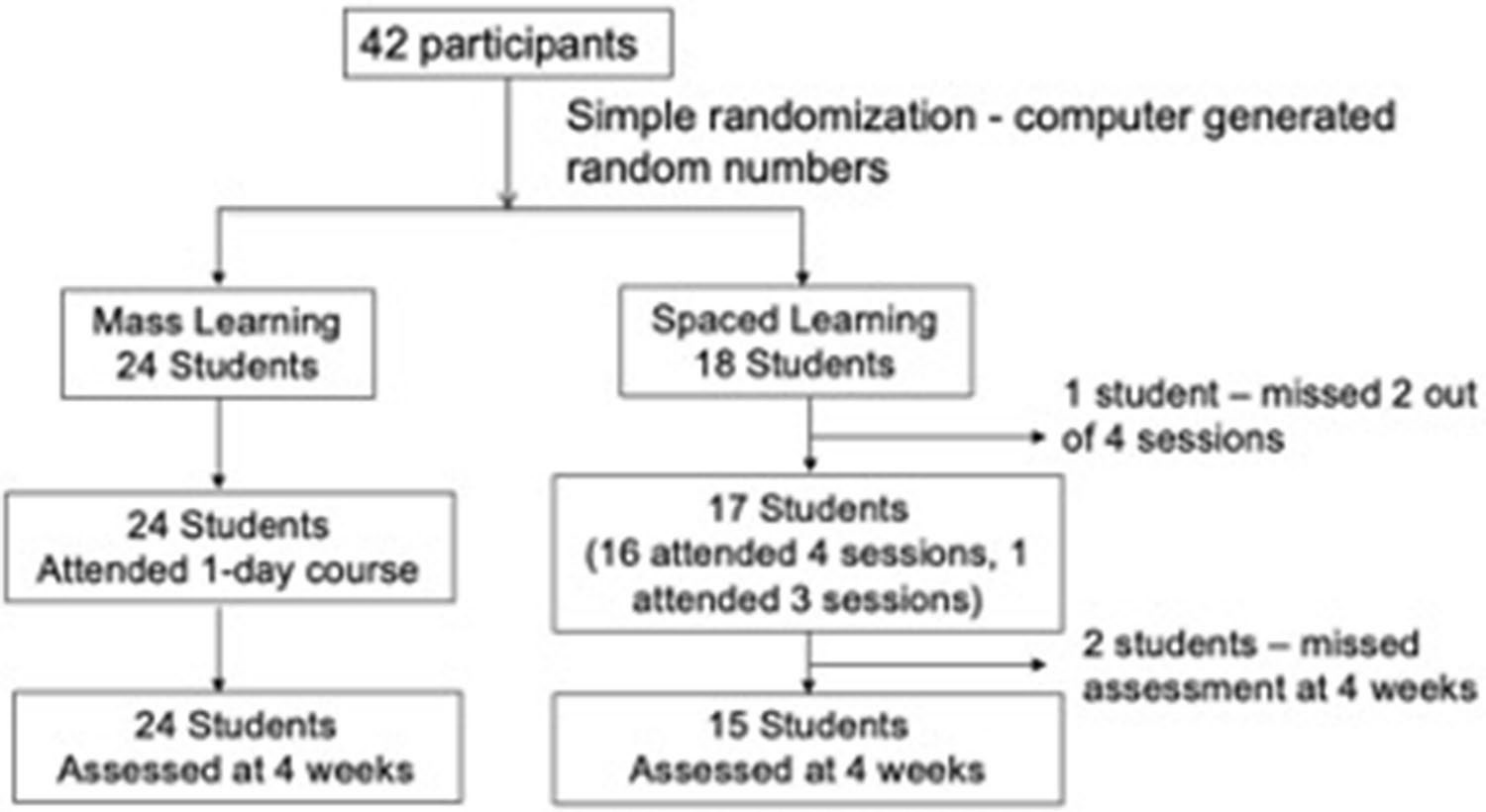
Overview of participant enrolment.

The participants who were randomly assigned to each group received the intended treatment with no cross-over and underwent per-protocol statistical analysis (see Fig. 1).

### Demographics of participants

The mean age, gender distribution, hand dominance and suturing experience of participants in both groups were similar with no statistical significance (see Table 1.) The mean age of participants was 22.38 years (SD = 0.87) in the massed learning group and 22.67 years (SD = 1.18) in the spaced learning group (t(37) = 0.89, p = 0.38). 58% of the participants in the massed learning group were male, compared to 46% in the spaced learning group (χ^2^(1, N = 39) = 0.51, p = 0.48). Both groups demonstrated comparable baseline suturing skills—total score of 27.50 (SD = 4.01) for mass learning group and 25.13 (SD = 8.48) in spaced learning group (t(37) = 1.49, p = 0.33).

**Table 1:** Baseline characteristics of participants.

Characteristics	Massed (n = 24)	Spaced (n = 15)	Test statistic	P value	Degrees of freedom
Age mean	22.4 (SD0.87)	22.7 (SD1.18)	0.89	0.38	
**Gender**					
Male	14 (58.3%)	7 (46.7%)	0.51	0.48	1
Female	10 (41.7%)	8 (53.3%)			
**Hand dominance**					
Right	22 (91.7%)	13 (86.7%)	0.25	0.62	1
Left	2 (8.3%)	2 (13.3%)			
**Prior suturing experience**					
Yes	3 (12.5%)	4 (26.7%)	1.26	0.26	1
No	21 (87.5%)	11 (73.3%)			
Strip 1 (total score)	27.50 (SD4.01)	25.13 (SD8.48)	1.49	0.33	

### Primary outcome

**Figure 2:**
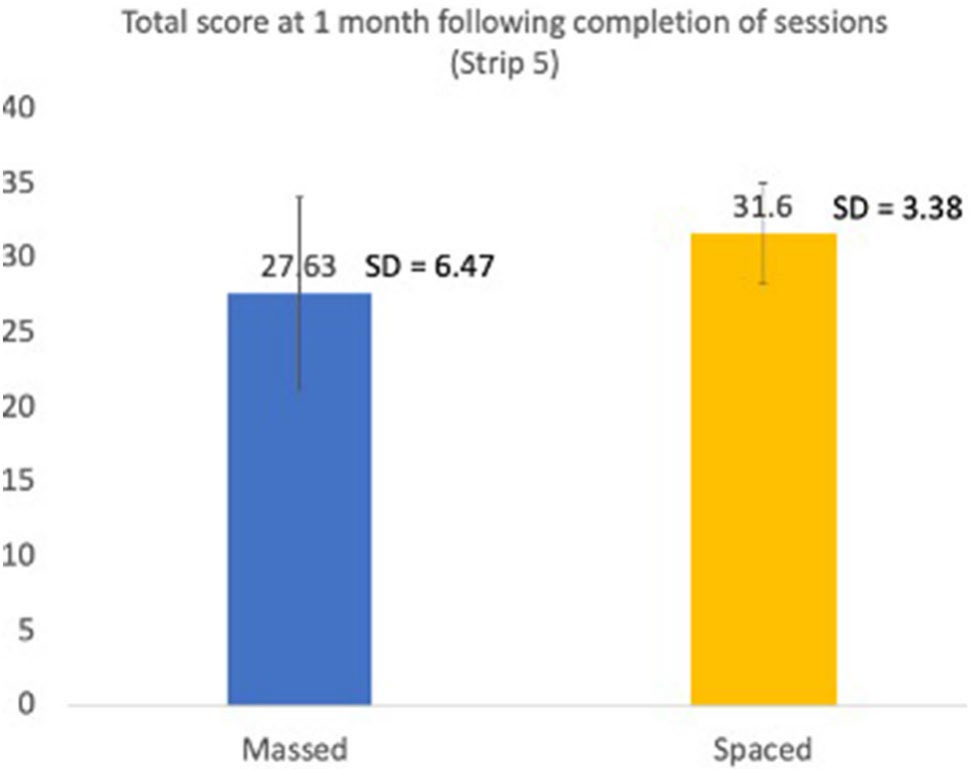
Primary outcome—total score at 1 month following completion of sessions (Strip 5).

The total score was higher in the spaced group compared to the mass learning group at 1 month after the completion of teaching sessions. The total score was 27.63 (SD = 6.47) in the mass learning group compared to 31.60 (SD = 3.38) in spaced learning group (t(37) = 2.19, p = 0.04) (see Fig. 2).

### Secondary outcomes

**Figure 3:**
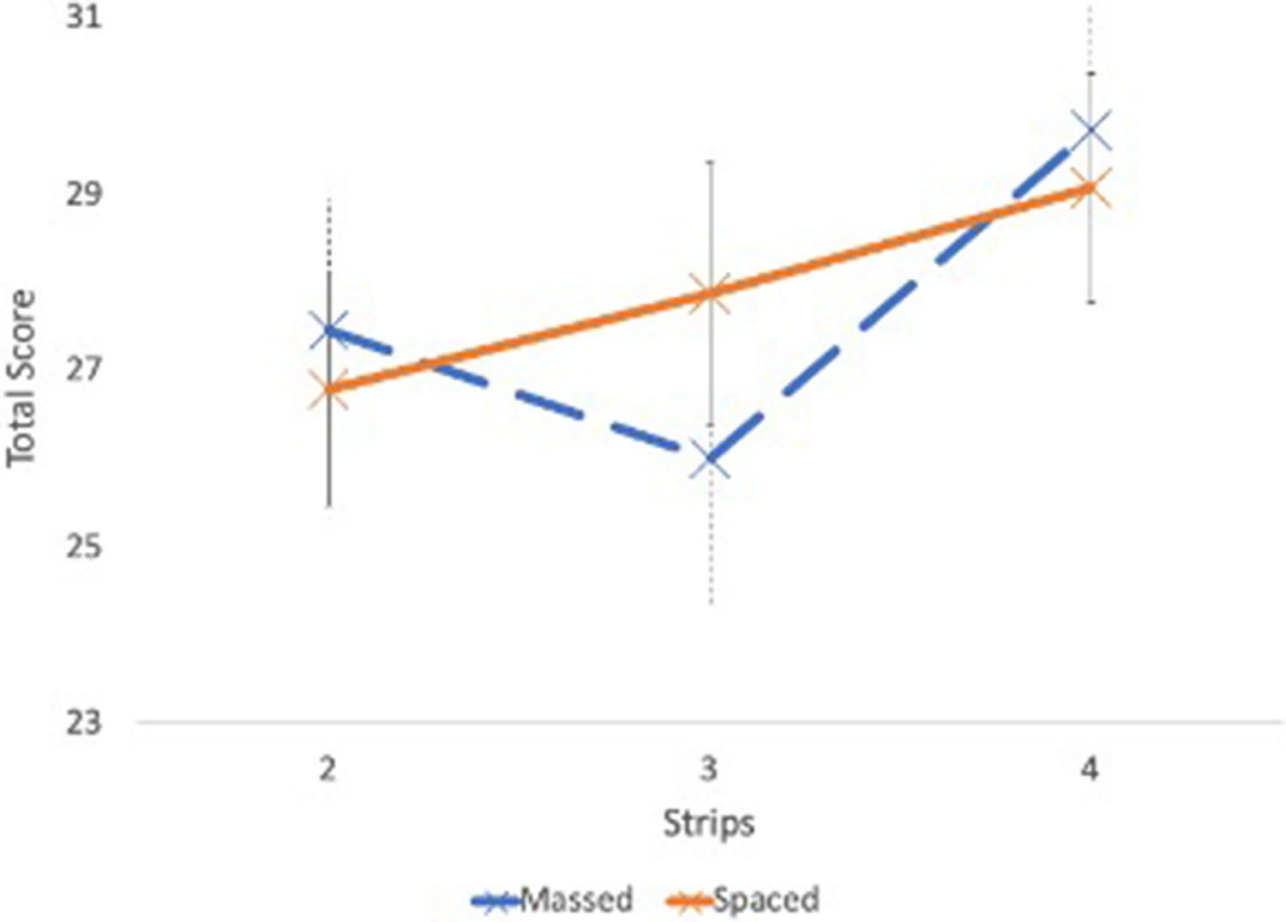
Mixed ANOVA test for Strips 2, 3 and 4 (Total Score).

There was no significant difference in the time taken to complete the test strip at 1 month after the completion of teaching sessions (strip 5) between both groups – 45.71 min (SD = 15.80) in mass learning group vs 44.07 min (SD = 13.20) in spaced learning group (t(37) = 0.34, p = 0.74). There was no difference in satisfaction scores between the two groups (8.00 vs 8.47, t(37) = 1.22, p = 0.23). The mixed ANOVA assessing the global performance of candidates during the training sessions (Strips 2, 3 and 4) indicated improvement in total scores over time F (2,46) = 3.29, p = 0.05 (see Fig. 3). There was no overall difference in scores between the mass and space learning groups over Strips 2,3 and 4 F (1,23) = 0.01, p = 0.92.

## Discussion

This study showed that spaced learning is superior to mass learning in acquiring microsurgical skills, with participants having better scores at 1 month after the completion of the teaching sessions. There was an overall improvement of scores during the training sessions in both groups but there was no global difference between the mass and space learning groups during this time. We conclude that regular training sessions led to improvement in microsurgical skills in both groups without significant differences between mass and space learning. However, space learning enhances *retention* of these complex skills over a longer period. Our findings are directly relevant to the training of microsurgeons. To attain proficiency in microsurgery, extensive training is necessary to gain the relevant motor and cognitive skills. A teaching model with increased effectiveness and efficiency not only reduces the total time spent to achieve proficiency but can also improve surgical outcomes over time if individuals are better able to retain skills. Satisfaction in both groups following the completion of sessions was also similar.

Our conclusions are supported by other studies. In 2006, Moulton et al. examined the spacing effect in acquiring skills for microvascular anastomosis with the use of Penrose drains, PVC arteries and arteries in turkey thighs^9^. In their study, the spacing effect was achieved by spreading out four training sessions over 4 weeks (one session per week), as opposed to the mass-learning group undergoing four training sessions in 1 day, and participants were assessed pretraining, immediately post-training, and 1 month post-training. The study also assessed the clinical transferability of skills acquired at the end of teaching with the use of live, anaesthetized rats^9^. The study involved 38 residents and assessed time taken, motion efficiency and general competency. They found that although both groups showed immediate improvement in performance, the spaced learning group demonstrated better retention of skills and also performed better than the mass learning group in the live rat anastomoses. The results of that study echoed our findings—both groups demonstrated improvement from baseline but the spaced learning group had better retention.

However, one of the strengths of our study that sets it apart from other studies is the use of a validated standardized software (Digital Surgicals Ptd Ltd) to objectively evaluate and assess the strips. This eliminates any possibility of assessment bias and strengthens the internal validity of the study. The removal of bias with the use of objective assessment tools consequently improves reproducibility, reliability, and validity of the results obtained. This study also differed from previous studies because we used flat strips for assessment and focused on the precise placement of sutures (deviation from wound edge and spacing of sutures placed) while other studies used Penrose drains, PVC (polyvinyl-chloride) tubes or animal vessels with assessment being based on global subjective ratings of performance^9,10^. The correct placement of sutures is an accurate reflection of the level of microsurgical skills acquired, as it is a product of precise control and fine hand–eye coordination.

The positive results of the spacing effect have been documented in studies carried out in other areas of surgery. Most notably, a recent systematic review examined spacing effect on different surgical skills (suturing and knot tying, laparoscopic skills, vascular anastomosis and microvascular anastomosis) and found that spaced learning improves short term and long- term surgical skills retention (between 5 min to 1 year)^4^. In addition to surgical skills, spacing effect has also been shown to bring about improved retention in medical education. A study investigating the spacing effect on online medical education in urology has found that the spacing effect persists up to 2 years^11,12^. Our results indicate a similar phenomenon with respect to microsurgical skills.

Our study has several limitations. There were a small number of participants, leading to insufficient power to detect smaller differences between the two groups. The primary outcome was assessed at 1 month after completion of the training sessions, and it is not known if the microsurgical skills would be retained over a longer period. Further investigations will also need to be done to elucidate the optimal gap between training sessions, which is an observation that has been echoed in previous studies^4^.

We conclude that that spaced learning aids in improved retention of microsurgical skills compared to mass learning. Our findings provide an impetus to implement alternatives in the way teaching courses are organized. Spaced learning is a flexible alternative that allows training sessions to be built into the hectic residency training routine and offers a viable alternative for microsurgical training that may improve the effectiveness and efficacy of teaching. The spacing phenomenon also mitigates the decay of skills, which is especially pertinent if participants are unable to achieve consistent exposure to microsurgery after the course. Our results also support an initiative to ensure surgical trainees routinely practice microsurgical suturing at regular intervals to improve currency and retention of this complex skill set. Such efforts are relatively inexpensive, because they do not require live animal models and may be used to maintain surgical competency even in the absence of clinical cases, such as during the current COVID pandemic^13^. The potential impact of the superior results witnessed with spaced learning also extends beyond microsurgical skills training; spaced learning can be adopted in undergraduate basic surgical skills training or in re-certification courses for surgeons.

## References

[1] Barsuk JH, Cohen ER, McGaghie WC, Wayne DB (2010). Long-term retention of central venous catheter insertion skills after simulation-based mastery learning. Acad. Med..

[2] Glenberg AM, Lehmann TS (1980). Spacing repetitions over 1 week. Mem. Cognit..

[3] Pashler H, Rohrer D, Cepeda NJ (2007). Enhancing learning and retarding forgetting: Choices and consequences. Psychon. Bull. Rev..

[4] Cecilio-Fernandes D, Cnossen F, Jaarsma DADC, Tio RA (2018). Avoiding Surgical Skill Decay: A systematic review on the spacing of training sessions. J. Surg. Educ..

[5] Spruit EN, Band GP, Hamming JF (2015). Increasing efficiency of surgical training: Effects of spacing practice on skill acquisition and retention in laparoscopy training. Surg. Endosc..

[6] Patocka C, Khan F, Dubrovsky AS, Brody D, Bank I, Bhanji F (2015). Pediatric resuscitation training-instruction all at once or spaced over time?. Resuscitation..

[7] Boettcher M, Boettcher J, Mietzsch S, Krebs T, Bergholz R, Reinshagen K (2018). The spaced learning concept significantly improves training for laparoscopic suturing: A pilot randomized controlled study. Surg. Endosc..

[8] Lahiri A, Sebastin SJ, Yusoff SK, Sze Chong AK (2016). Computer aided assessment in microsurgical training. J. Hand. Surg. Asian Pac..

[9] Moulton CA, Dubrowski A, Macrae H, Graham B, Grober E, Reznick R (2006). Teaching surgical skills: What kind of practice makes perfect?: A randomized, controlled trial. Ann Surg..

[10] Mitchell EL (2011). Evaluation of distributed practice schedules on retention of a newly acquired surgical skill: A randomized trial. Am. J. Surg..

[11] Kerfoot BP (2009). Learning benefits of on-line spaced education persist for 2 years. J. Urol..

[12] Kerfoot BP, Fu Y, Baker H, Connelly D, Ritchey ML, Genega EM (2010). Online spaced education generates transfer and improves long-term retention of diagnostic skills: A randomized controlled trial. J. Am. Coll. Surg..

[13] De Das S, Puhaindran ME, Sechachalam S, Jian KHW, Chew WC, Yuan AHC (2020). Sustaining a national surgical training programme during the COVID-19 pandemic. Bone Jt. Open..

